# One test, many tongues: Surveying language proficiency across the globe

**DOI:** 10.1073/pnas.2420179123

**Published:** 2026-03-27

**Authors:** Pol van Rijn, Yue Sun, Harin Lee, Raja Marjieh, Ilia Sucholutsky, Francesca Lanzarini, Elisabeth André, Nori Jacoby

**Affiliations:** ^a^Max Planck Institute for Empirical Aesthetics, Computational Auditory Perception Research Group, Frankfurt am Main 60322, Germany; ^b^Deutsche Bundesbank, Research Data and Service Centre, Frankfurt am Main 60431, Germany; ^c^Cooperative Brain Imaging Center, Goethe University, Frankfurt am Main 60590, Germany; ^d^Ernst Strüngmann Institute for Neuroscience, Frankfurt am Main 60528, Germany; ^e^Max Planck Institute for Human Cognitive and Brain Sciences, Leipzig 04103, Germany; ^f^Princeton University, Department of Psychology, Princeton, NJ 08544; ^g^University of Augsburg, Chair for Human-Centered Artificial Intelligence, Augsburg 86159, Germany; ^h^Cornell University, Department of Psychology, Ithaca, NY 14850

**Keywords:** online experiments, vocabulary test, lexical decision, cross-cultural psychology, language proficiency

## Abstract

Language influences our thinking and affects many aspects of cognition, from how we perceive the world to how we interact socially. Thus, objectively characterizing linguistic background is crucial for research in many areas, including second language acquisition, psycho-linguistics, and cognitive science. Traditional language proficiency tests, however, are manually composed by experts, limiting their scope for both lab and online settings. Here, we propose a pipeline that automatically derives a language proficiency test from a corpus of text and applies it to create new tests for 1,939 languages. Using this approach, we conducted a large-scale survey examining L1 and L2 proficiency across 34 countries, with participants tested on all 34 languages. Drawing from human ratings from 4,137 participants, our results validate that our test can effectively distinguish native speakers, second-language speakers, and nonspeakers within one minute, making it an effective tool for evaluating linguistic proficiency. We show that participants’ linguistic and demographic backgrounds systematically influence both their language proficiency and their self-reported skills, and we map the prevalence of global languages, such as English and Spanish, among online participants. Moreover, we show that our vocabulary tests are strongly correlated with other linguistic competences—such as listening and writing—in a set of typologically varied languages, demonstrating our test is an efficient instrument to assess language proficiency. More broadly, our work offers a significant resource for investigating global variation in language skills and contributes to reducing the overreliance on the English language in the cognitive and social sciences.

## Significance

Measuring language proficiency is essential for research in many areas, including second language acquisition, psycholinguistics, and cognitive science. We propose a method to derive language proficiency tests from texts and apply it to generate new tests for 1,939 languages. We extensively tested this through experiments with 4,137 participants. We used the method to test the linguistic background of speakers in their first and second language in 34 languages across 34 countries and characterize how our test is influenced by linguistic and demographic factors. Overall, our work provides a complementary tool for assessing global variations in language proficiency, offering an alternative to existing approaches and helping to reduce the field’s overreliance on the English language in the cognitive and social sciences.

The language we speak profoundly affects our thoughts, beliefs, and concepts (1). Language and cultural background affect cognitive abilities not only for language-related tasks, such as emotion semantics (2), but also for ostensibly nonlinguistic abilities, including memory (3), space (4), time (5, 6) and sensory-perception (7–10). Thus, determining a participant’s linguistic background is crucial for research in many areas, including second language acquisition (11), psycholinguistics (12, 13), neuroscience (14, 15), and cognition (16). With more than 7,000 languages spoken worldwide (17), our species exhibits a strong linguistic diversity (18). However, research in these areas mostly involves English-speaking participants recruited from Western, Educated, Industrialized, Rich, and Democratic societies (WEIRD; 19, 20). This restricts the generalizability of the findings on human cognition and behavior (7).

With increased global access to the internet, it has become possible to recruit online participants from all over the world (21–26). However, online participants may have diverse multilingual backgrounds (27), may provide noisy responses (28, 29), and can be less motivated or honest than lab participants (30). This highlights the need for objective measures to assess participants’ first and second language skills beyond subjective measures such as self-reports (31).

Vocabulary knowledge and proficiency have traditionally been assessed using tools such as Nation’s Vocabulary Levels Test (VLT) (33, 34), which gauges recognition of words from graded frequency lists, as well as oral methods such as sentence repetition tests that measure bilingual proficiency through recall of increasingly complex sentences (35) and structured oral interviews or imitation tasks (36). While effective within their domains, these approaches rely on fixed item lists or expert-led administration, limiting their scalability and adaptability to diverse languages. Recent studies have refined these methods—Ha (37) highlights the VLT’s broad utility as a vocabulary benchmark, and Milliner (38) introduced an online platform for customizable VLTs-yet they remain constrained by predetermined content.

Within this broader tradition of vocabulary-based proficiency testing, LexTALE (32) has emerged as a widely used and time-efficient alternative. LexTALE operationalizes vocabulary knowledge through a lexical decision task in which participants identify real words from lists that also contain pseudowords. Test items are typically constructed by manually selecting rare real words and generating structurally similar pseudowords, resulting in a task that native speakers find easy and nonnative speakers find more difficult. The effectiveness of lexical decision tasks as indicators of language proficiency is further supported by recent evidence (39) showed that lexical decision measures, including LexTALE, were among the strongest predictors of English proficiency across first- and second-language speakers from different L1 backgrounds. While initially developed for English, LexTALE has since been extended to 13 additional languages (40–51).

However, LexTALE is limited in its generalizability. Manually creating the word list requires human domain experts and, therefore, introduces subjective biases. This reliance on human labor also limits the number of words in the list, which restricts the possibility of repeated testing. Furthermore, LexTALE requires a word frequency database that is not available for low-resource languages. In fact, LexTALE is currently available in 14 languages, while in comparison, online recruitment platforms can provide access to speakers of more than 90 languages (52, 53).

**Fig. 1.**
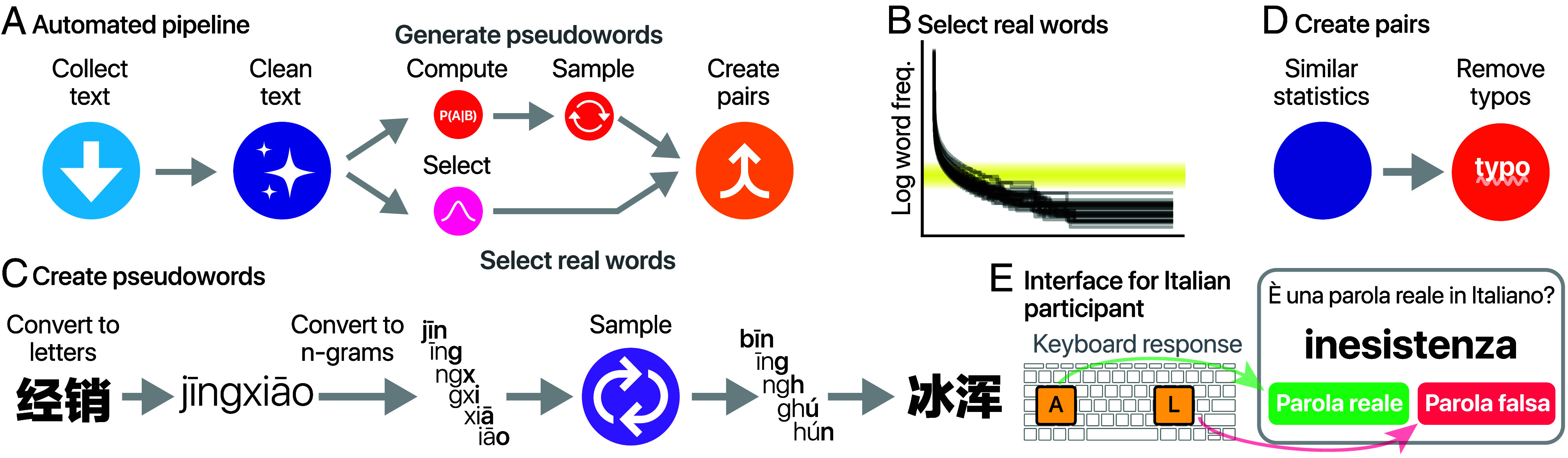
Vocabulary test generation. (*A*) Summary of the automated pipeline consisting of the following steps: collecting and cleaning the text, creating pseudowords, selecting rare words, and matching the real and pseudowords. (*B*) To establish a consistent test difficulty, we computed the abundance of words with corpus and selected word frequencies that were similar to the one used in previous nonautomatic tests (32) (shaded yellow area). (*C*) Creating pseudowords by computing the n-gram transitional probabilities of letters within words and drawing samples from the transitional probability. For illustration purposes, we display 3-g (in the pipeline, we used 5-g). Character-based languages like Chinese are first converted into letter-based representations (e.g., Pinyin) to compute the transitional probabilities. The letters are later converted back to character sequences. (*D*) Real and fake words are paired based on their low-level statistics. We remove pairs where the pseudoword is too close to one word in the dictionary, as it might be read as a typo of a real word. (*E*) Interface of the validation experiment for an Italian participant tested in Italian. The word is presented as an image to avoid copy-pasting.

In order to overcome these limitations, we developed a completely automated pipeline for creating language proficiency tests for any language (Fig. 1*A*).^*^ In its core, our pipeline requires only a text corpus of a given language but can take advantage of additional resources (such as online dictionaries) if they are available. Rather than aiming to model all language-specific grammatical or semantic idiosyncrasies, the pipeline is explicitly designed to be as generalizable across languages, even at the cost of occasional imperfections in individual cases. In this study, we applied the pipeline to two text sources: Wikipedia articles for 60 languages (WikiVocab) and the text of the Bible translations in 1,939 languages (BibleVocab). This significantly expanded the number of languages in which we can conduct a language proficiency task. With BibleVocab, we can theoretically identify native speakers in over 6.3 billion speakers from over 200 countries (54) (*SI Appendix*, section L). The pipeline automatically identifies difficult words real words based on their frequencies of occurrence within the text corpus (Fig. 1*B*) and creates pseudowords by sampling from the transitional probabilities of the letter n-grams of real words (Fig. 1*C*). Real and fake words are paired according to the transition probabilities of the letters and thus present similar structure (Fig. 1*D*, see *SI Appendix*, Table S1 for example items). While this fully automated approach may not capture all language-specific phenomena (e.g., particular function words or morphological constructions in some languages), it provides a consistent and extensible foundation that can be refined with additional heuristics where needed. The resulting tests^†^ can be conducted both in the lab and online (Fig. 1*E*).

We extensively tested our pipeline, conducting large-scale online experiments. In the first experiment, we applied our pipeline to Wikipedia articles and conducted vocabulary test on participants on Prolific (52), a common online recruitment platform. We show that the generated test can accurately identify native speakers, and separate them from nonnative speakers of closely related languages. We then replicate the findings on Cint Marketplace—another recruitment platform which is used for marketing services and has much wider global reach but is less used in cognitive, psycholinguistics, and social science.

Overall, we utilized the pipeline to conduct language proficiency test in 34 countries, assessing proficiency in each country across all 34 languages. We show how language proficiency is predicted by participants’ demographics, linguistic distance to the native language of the participant, and self-reported language proficiency. We also compare the prevalence of global languages like English and Spanish with national languages such as Greek and Vietnamese. This comparison provides valuable insights into the linguistic landscape, showing how some languages have a global reach, while others are mainly used locally, and contributes to understanding language spread and cultural influence.

Furthermore, to test the generalizability of our pipeline, we applied it to the Bible and provided vocabulary tests for 1,939 languages (BibleVocab). We show that the resulting tests, while created on smaller text corpora and minimal preprocessing, can still distinguish between native speakers in typologically related languages. This finding demonstrates that the pipeline can be applied to an open-ended number of languages, even to those with relatively low resources. Finally, we show that the vocabulary test performance is strongly correlated with other linguistic competences—such as comprehension and writing—in four typologically varied languages, indicating that our test is a good proxy for other language proficiency skills. Taken together, our pipeline facilitates global recruitment for large-scale, multilingual research programs by offering a scalable method to study language skills across diverse populations and provide insights into language proficiency in 34 countries.

## 1. Results

### 1.1. Experiment 1: Vocabulary Tests Distinguish between Closely Related Languages on Two Recruitment Platforms.

We developed vocabulary tests for 60 languages based on Wikipedia (*SI Appendix*, section C and Fig. 1*A*). With more than 300 languages available, Wikipedia is one of the largest collaborative internet projects, allowing us to estimate vocabulary frequency in a diverse set of languages. In addition to basic, language-independent word cleaning processes, such as eliminating words containing numbers (*SI Appendix*, section E), we utilized online dictionaries to identify misspelled words and employed natural language processing techniques to exclude proper nouns (*SI Appendix*, section F). These resources are accessible in the 60 Wikipedia languages, and our findings indicate that they enhance the overall quality, although they are not crucial (*Experiment 3*).

From the cleaned words, we created matched nonlexical items (pseudowords) by sampling from the transitional probabilities of the letter n-grams (*SI Appendix*, section H and Fig. 1*C*). We identify rare words based on the frequency of occurrence in the corpus (*SI Appendix*, section I and Fig. 1*B*). Changing the frequency of the selected words alters the difficulty of the test. To identify proficient speakers, we selected words with a mean frequency of 10 occurrences per million, comparable to previous LexTALE tests (32). We also developed automated solutions to deal with edge cases, including languages with compound words (*SI Appendix*, section E) and character-based languages (such as Chinese, Korean, and Japanese; *SI Appendix*, section F). Each participant receives a list of real and pseudowords and is asked to mark whether the presented word is real or fake (*SI Appendix*, section K and Fig. 1*E*).

**Fig. 2.**
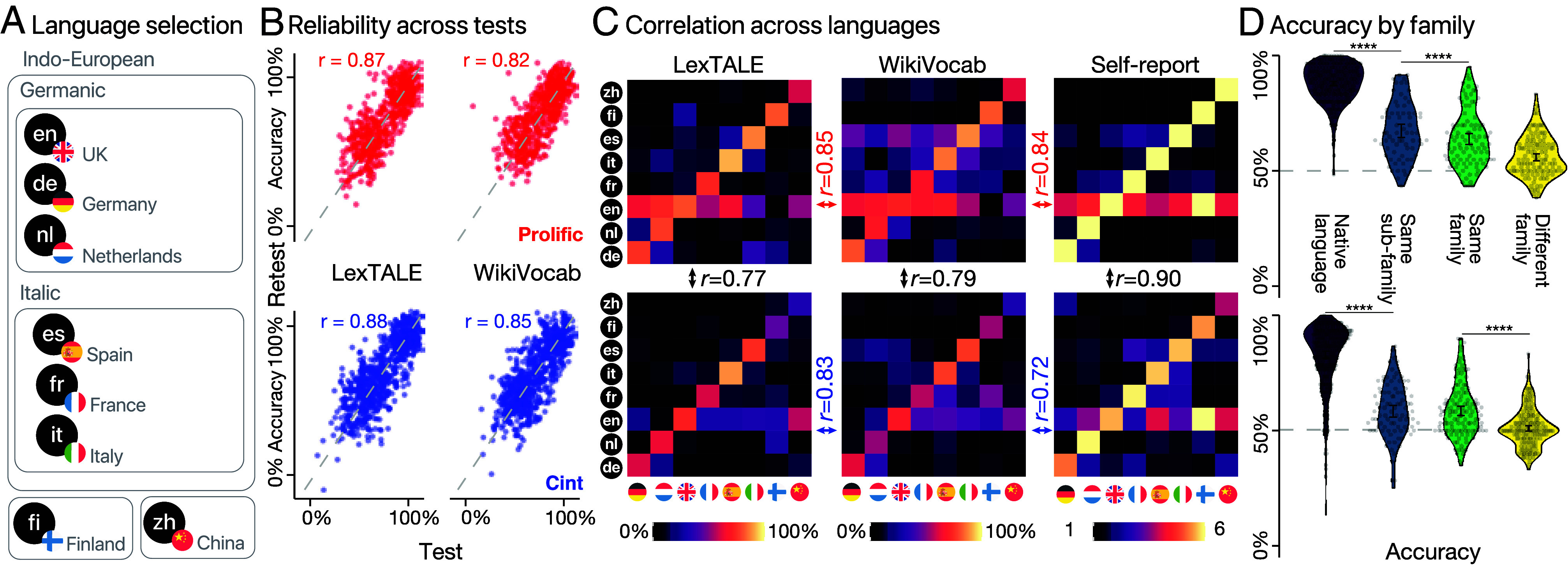
(*A*) Selected typologically different languages used in a fully crossed study design. At least 35 monolingually raised participants from the United Kingdom, Germany, the Netherlands, Spain, France, Italy, China, and Finland participate in a language test in their native (L1) and in a randomly selected foreign language (L2) recruited from Prolific (*SI Appendix*, Table S2) and Cint (*SI Appendix*, Table S3). (*B*) Test–retest reliability (Pearson correlation) between two blocks for WikiVocab and LexTALE in both recruiters. (*C*) Average performance score for LexTALE, WikiVocab, and self-report in Prolific (*Upper* panel) and Cint (*Lower* panel). The x-axis represents the eight countries, and the y-axis the eight languages. The color-fill indicates the accuracy. Vertical Pearson correlations reflect the correlations of the same test in different recruiters. Horizontal correlations show the correlation between WikiVocab and LexTALE and self-report within the same recruiter. (*D*) Violin plot of the WikiVocab test performance for native language, same subfamily, same family, or different family. Dots represent the test scores of single participants. The error bar is the SEM. ****P< 0.0001.

To assess the quality of our test, we benchmark it with eight existing LexTALE tests (32, 41, 44–46, 50) and examine whether the test would be able to distinguish between native speakers of closely related languages. We selected eight languages that include both linguistically distant pairs (e.g., French and Chinese) and closely related ones (e.g., Dutch and German). Six languages are Indo-European languages including three Germanic languages (English, German, Dutch) and three Italic languages (Spanish, French, Italian). In addition, we also add two typologically distinct languages (Chinese and Finnish, Fig. 2*A*, Section 3.2). In total, we recruited 303 participants from Prolific (Sections 3.1 and 3.2) and compared participants’ performance in WikiVocab to LexTALE. Participants were tested on their self-reported native language and one additional language selected randomly from the seven remaining languages. The entire test was repeated twice (with different items) such that we could assess test–retest reliability. In addition, participants provided self-reported language proficiency in all eight languages.

Fig. 2*B* shows that both WikiVocab and LexTALE were highly reliable, the correlation between the performance in the first and the second block for WikiVocab (*r* = 0.82 [0.78, 0.85], *P*< 0.001) was nearly as high as the one for LexTALE (*r* = 0.87 [0.85, 0.89], *P*< 0.001). The performance of the two tests was highly correlated (*r* = 0.85 [0.83, 0.88], *P*< 0.001). The heatmaps in Fig. 2*C* depict the performance on LexTALE, WikiVocab, and the language self-report. For both vocabulary tests, there is a prominent diagonal, indicating higher accuracy in participants’ native language compared to the other languages. The average performance on the main diagonal was 88 [87, 89]% in WikiVocab and 89 [88, 90]% for LexTALE, whereas on all other languages, it was 62 [60, 63]% in WikiVocab and 57 [55, 58]% for LexTALE, this difference was significant for both WikiVocab (*d* = 2.6) and LexTALE (*d* = 3.2; see Fig. 2*D*). This indicates that both tests effectively distinguish native from nonnative speakers of a language, with LexTALE yielding slightly higher scores for natives and slightly lower scores for nonnatives. Importantly, as shown in Fig. 2*D*, participants’ performances in their native language was higher than in the other languages in the same subfamily (WikiVocab: 68 [65, 70]%, LexTALE: 62 [58, 65]%). Their performance dropped further for language in the same broader family (WikiVocab: 64 [62, 66]%, LexTALE: 57 [55, 60]%) and their performance for languages from different families approached chance level (WikiVocab: 56 [55, 58]%, LexTALE: 53 [52, 55]%).

The test performance was correlated with self-reports for WikiVocab (*r* = 0.84 [0.82, 0.86], *P*< 0.001). Interestingly, all matrices show a horizontal line for English, indicating that most participants are quite fluent in this language. Overall, in terms of performance, LexTALE slightly outperformed WikiVocab, but considering that WikiVocab was created through an automated procedure, it is quite remarkable that it achieved almost the same high level of performance without utilizing domain experts’ knowledge.

**Fig. 3.**
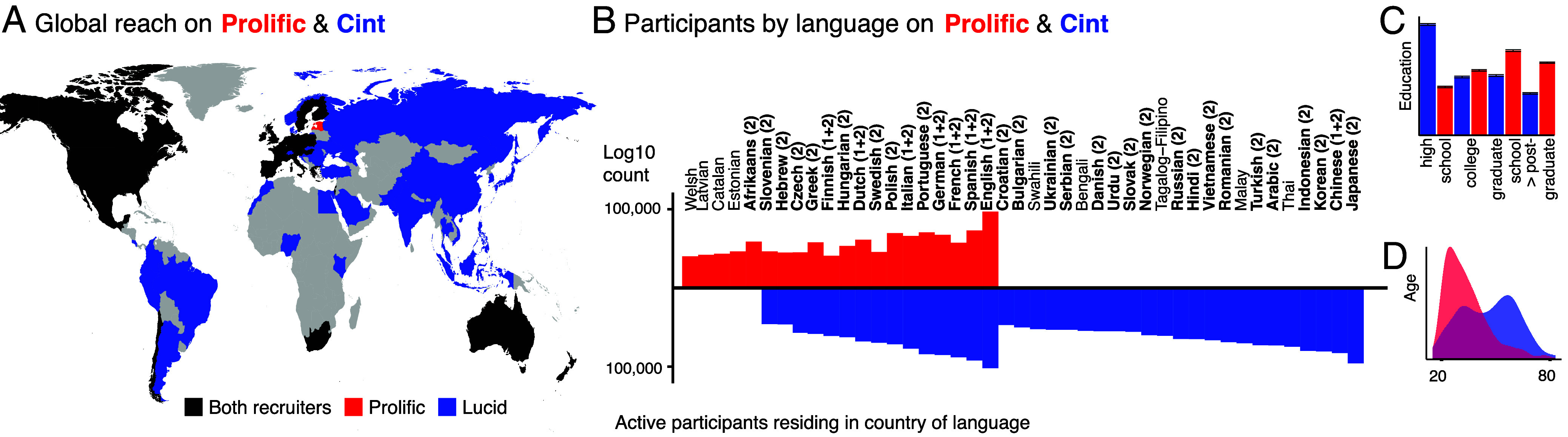
Recruiter differences on Prolific and Cint. (*A*) Recruitable countries in Prolific (red), Cint (blue), and both (black). (*B*) Number of participants per language on Prolific and Cint. For a country or language to be listed, it must have at least 100 active participants. We mark the countries recruited in experiment 1 with “1” and those recruited in experiment 2 with “2.” Demographic differences on Prolific and Cint (experiment 1): (*C*) Education and (*D*) Age.

While Prolific is commonly used to recruit participants in the cognitive and social sciences (55), it has a rather limited global reach, with most of the recruitable participants located in the United States and in Europe (Fig. 3*A*). The number of languages one can recruit from is also limited (Fig. 3*B*). Therefore, we use Cint as an additional recruiting platform to test the vocabulary test for a wide array of languages and replicate the previous findings on Cint (*N* = 430, see *Lower* panel of Fig. 2*B*). Performance in the two tests was comparable to Prolific (see *Lower* panels of Fig. 2*B*–*D*). The test–retest reliability was slightly higher on Cint (LexTALE: *r* = 0.88 [0.86, 0.9], WikiVocab: *r* = 0.85 [0.83, 0.87]) than on Prolific.

The heatmaps in Fig. 2*C* show a similar structure of performance compared with Prolfic (LexTALE: *r* = 0.77 [0.68, 0.84], WikiVocab: *r* = 0.79 [0.71, 0.86], Self-report: *r* = 0.90 [0.84, 0.93]). For both vocabulary tests, there is a prominent diagonal (average performance on the main diagonal; WikiVocab: 83 [81, 84]% LexTALE: 85 [83, 86]%), indicating higher accuracy in participants’ native language (WikiVocab: *d* = 2.1, LexTALE: *d* = 2.3) compared to nonnative languages (average performance off-diagonal; WikiVocab: 55 [54, 57]%, LexTALE: 54 [53, 55]%). Further assessments revealed a similar performance pattern as shown in participants from Prolific: Participants performed best in their native language, exhibit increasingly lower performances in nonnative languages from the same subfamily (WikiVocab: 59 [56, 61]%, LexTALE: 56 [54, 58]%) and broader family (WikiVocab: 59 [57, 61]%, LexTALE: 54 [52, 56]%), and yielded near chance level performances in languages from a different language family (WikiVocab: 51 [50, 52]%, LexTALE: 53 [51, 54]%) (Fig. 2*D*).

The results on Cint differ from Prolific in two ways: the diagonal is slightly weaker on Cint than on Prolific—indicating overall lower scores in the native language of participants on Cint—and the horizontal line for English is not as strong as on Prolific, indicating lower English proficiency for participants on Cint (Cint: 68.5 CI = [62.2, 74.7]%, Prolific: 77.6 CI = 69.3, 86.0]%). These differences suggest that Cint participants capture a much wider performance diversity, particularly including participants with lower English performance than Prolific. This is likely due to a higher degree of socioeconomic diversity and lesser exposure to English. Indeed, participants on Prolific had an overall higher formal education (bootstrapped, percentage highest degree is postgrad or higher, Cint: 15.5%, Prolific: 26.2%, *P*< 0.001) and covered a smaller age span on average (bootstrapped, mean age span on Prolific: 41.5 y, Cint: 53.5 y, *P*< 0.001, see *Difference between Prolific and Cint Recruiters* in *SI Appendix*), as well as had larger average degree of self-reported proficiency in English (Self-report ranges from 1 to 6; Prolific average: 5.2 [5.0, 5.6]; Cint average 4.8 [4.5, 5.1], difference: *d* = 3.2).

### 1.2. Experiment 2: A Survey of L1 and L2 Proficiency Across 34 Countries, with 34 Languages Tested in Each Country.

**Fig. 4.**
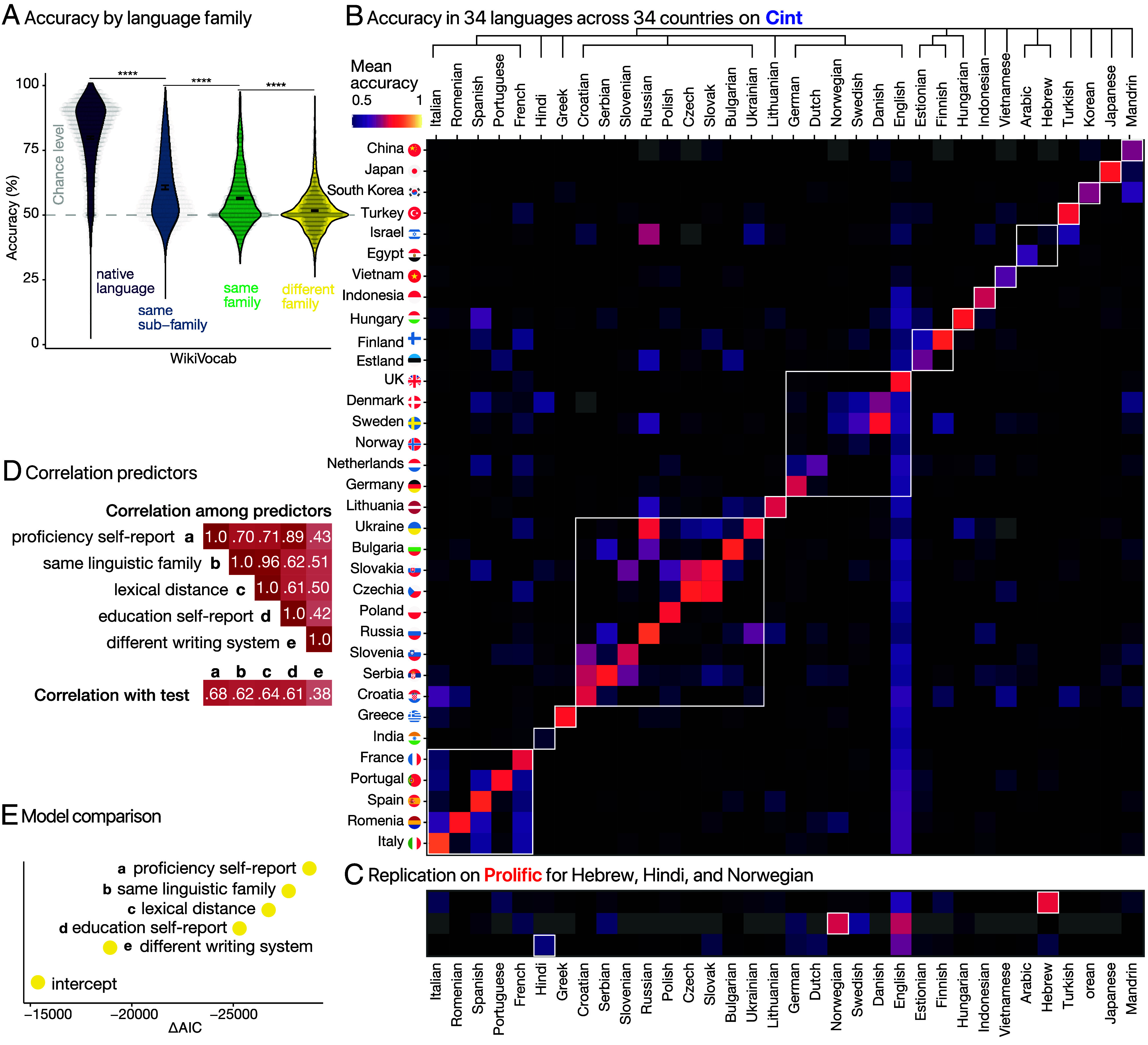
Extended testing on 34 languages. (*A*) Violin plot of accuracy in the test for different language comparisons. The same type of plot as in Fig. 2*D*. ****P< 0.0001. (*B*) WikiVocab accuracy for 34 × 34 languages on Cint. Languages are sorted by lexical distance (59). The tree indicates the language family and subfamily. (*C*) Replication of languages with a weak diagonal on Prolific. (*D*) Correlation between predictors and test score. (*E*) Model comparison.

Having established that our test can differentiate native speakers of typologically related languages, we extended our methodology to a broader range of languages to evaluate its applicability on a significantly larger scale. We selected 34 languages with a sufficiently large pool of active participants on Cint (Fig. 3; see *Difference between Prolific and Cint Recruiters* in the *SI Appendix*) spanning nine different writing systems (e.g., Cyrillic, Devanagari, Korean) and 15 language subfamilies (e.g., Slavic, Malayo-Polynesian, Indo-Iranian). We tested participants in their native language and three randomly chosen languages (prioritizing foreign languages spoken by participants; Methods 3.3). Consistent with Experiment 1, participants yielded best performance in their native language (79.2%), and progressively lower performances in nonnative languages from the same subfamily (60.4%; *d* = 1.40), and in those from the same broader family (56.4%, *d* = 1.80), and exhibited near chance level performances in languages from a different language family (54.5%, Fig. 4*A*). For instance, on average, a native German speaker doing the Dutch test (same language subfamily) should perform better than a Spanish native speaker doing the Danish test (same family), and participants doing a language from a different family should do even worse (e.g., an Arabic native speaker doing the Chinese test). Fig. 4*B* shows that most languages exhibit a prominent diagonal, indicating that participants performed best in their first language (L1) compared to any second language (L2), with a large effect size (*d* = 1.94). However, for Hebrew, Hindi, and Norwegian, the diagonal is less pronounced, suggesting that the performance gap between L1 and L2 is relatively small.

To validate the effectiveness of our tests for these three languages, we conducted follow-up experiments with native speakers recruited on Prolific. We also changed the experiment so that the participants were not required to reside in the corresponding countries, to have a wider range of participants included in the experiment. As shown in Fig. 4*C*, Hebrew and Norwegian speakers demonstrated higher performance in their respective L1s compared to L2s (Hebrew: L1 = 81 [79, 83]%, L2 = 56 [55, 58]%, *d* = 2.30; Norwegian: L1 = 80 [76, 83]%, L2 = 67 [63, 71]%, *d* = 1.05). For Hindi, the pattern was similar to what we observed on Cint: participants performed best in English (73 [70, 77]%) followed by Hindi (61 [58, 64]%).

A likely explanation for the Hindi results is the high level of multilingualism among Hindi speakers. In fact, on Prolific, we were unable to recruit participants who were raised as monolingual Hindi speakers. This observation suggests that the conceptualization of language proficiency in these participants may differ from speakers raised in more monolingual environments (54, 56).

The relatively high performance of Israeli participants on the Russian test in Cint may be attributable to the significant wave of immigration from Russia to Israel in the 1990s (57) [approximately 12.4% of Israelis are Russian-speaking (58)]. The absence of this effect on Prolific-despite generally high consistency across platforms (Fig. 2*B*)—may reflect demographic differences between participant pools (Fig. 3); however, from our data, we cannot rule out the possibility that the test is specifically less effective in Hebrew.

For Norwegian, we repeated the Cint experiment approximately 6 mo later. The new cohort outperformed the previous one on the same L1 test (73 [71, 76]%, *d* = 1.16 vs. 56 [53, 58]%, *d* = 0.36), indicating variability in participant characteristics over time on Cint. This fluctuation is consistent with the observation that those languages have the weakest correlations between self-reported language proficiency and test performance (*SI Appendix*, Fig. S1*A*), and that these three languages exhibit low test-retest reliability (*SI Appendix*, Fig. S1*B*). Together, these findings underscore the importance of objectively measuring language proficiency rather than relying solely on self-reports, and that the effectiveness of the test should be examined empirically. Given that we have evidence that the test is effective in nearly all the languages we tested, we next examined how participants performed across all language tests, focusing on asymmetries and mutual intelligibility between closely related languages.

First, we note that in some cases, performance is symmetric (Fig. 4*B*). For example, Slovakian participants performed well in Czech (a linguistically close language) (79 [74, 84]%) and vice versa (83 [80, 86]%). Similarly, Russians understand Ukrainian (72 [68, 77]%), and Ukrainians understand Russian (82 [75, 87]%). A similar pattern is found for Serbian speakers, who scored well in both Slovenian (73 [67, 80]%) and Croatian (78 [67, 87]%) relative to their native language (85 [83, 87]%). However, not all relations are symmetrical. For example, Portuguese participants perform better in the Spanish test (64 [59, 69]%) than Spanish participants did on the Portuguese test (56 [50, 62]%).

To investigate the contribution of different linguistic and demographic factors to test performance, we fitted linear mixed effects models to predict the accuracy of the participants using one of six predictors. The predictors are a) language proficiency from self-report, b) typological categorization of the language as used in Fig. 4*A*, c) lexical distance between native and tested language (59), d) years of formal education in the tested language, and e) whether the tested writing system differs from the writing system in the native language. Since the predictors are strongly correlated with each other (Fig. 4*D*), e.g., the self-reported language proficiency is strongly correlated with the number of years of language education (*r* = 0.89 [0.89, 0.90]), we fit seven independent models where each model only uses one predictor and the last model contains only the intercept without any predictor. Each model estimates a random effect for each participant and test block. As shown in Fig. 4*E*, the accuracy is best predicted by the self-report of the participants (ΔAIC: 0), followed by the linguistic classification (ΔAIC: −1,021), lexical distance (ΔAIC: −2,010), and years of formal education (ΔAIC: −3,442). The model using the writing system (ΔAIC: −9,816) to predict test accuracy performed substantially worse.

Overall, these results show that while several linguistic and demographic factors influence performance, self-reported language proficiency is the most reliable predictor, surpassing structural language relationships and formal education in explanatory power.

However, in rare cases—such as Russian-speaking participants in Israel or the initial Norwegian cohort on Cint—self-reported proficiency diverged from actual test performance, underscoring the importance of incorporating objective measures of language ability and testing the effectiveness of the test experimentally.

### 1.3. Experiments 3 and 4: Assessing the Robustness of the Findings.

Having shown that our pipeline works well for a large array of typologically diverse languages, the question remains if it works equally well on other corpora. Specifically, Wikipedia covers a limited number of languages, primarily those that are widely spoken and, consequently, have more resources, such as dictionaries, available. Moreover, the overall amount of text from Wikipedia tends to be very large for the selected languages, which is not the case for all languages. To address these issues, we apply our pipeline to 1,939 Bible translations.^‡^ In contrast to WikiVocab, we only applied basic preprocessing to text materials (*SI Appendix*, sections *E* and *G*). The other steps of the pipeline remained identical: we identified real words with low frequencies of occurrence in the corpus. We created matching pseudowords by sampling from the transitional probabilities of the letter n-grams. Finally, we matched pseudowords and real words with similar n-gram transitional regularities to obtain a final list of test items (Fig. 1).

**Fig. 5.**
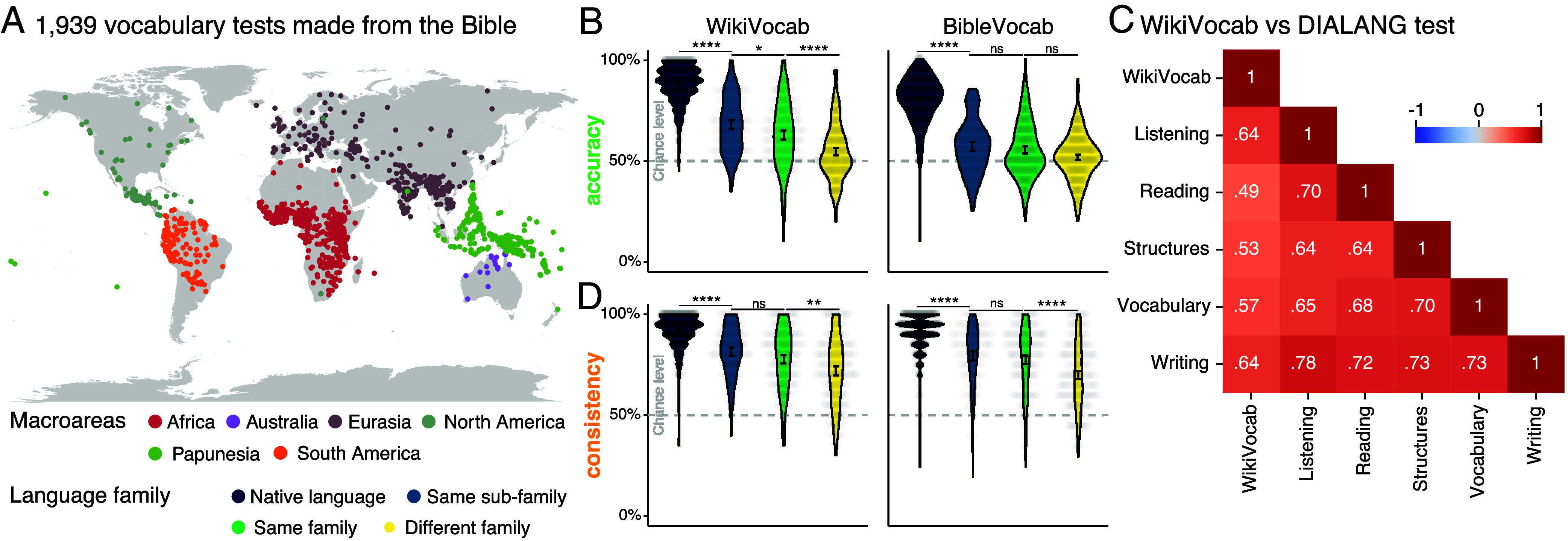
Assessing the robustness of the findings. Implementation of BibleVocab available in 1,939 languages (*A*). (*B*) Violin plot of accuracy in Bible- and WikiVocab for native language, same subfamily, same family, or different family. ns = *P*> 0.05, *P< 0.05, **P< 0.001, ****P< 0.0001 (*C*) Compare WikiVocab against a test battery assessing various linguistic skills.

To assess the comparability between BibleVocab and WikiVocab, we recruited additional 322 participants in experiment 3 from Prolific with speakers of English, Dutch, German, French, Spanish, Italian, Finnish, and Chinese (Section 3.4). To compare the performance of the tests, participants performed two blocks of WikiVocab and BibleVocab in their native language and a random second language. However, this time, they only did 20 items per block, and this time, they were repeated at the end of each block to measure item-level consistency. Similarly to the WikiVocab results from experiment 1 (*d* = 2.1), we found that the performance in L1 is higher than all other L2s (*d* = 2.08, see Fig. 5*B*). We also found that the accuracy on BibleVocab is significantly higher in L1 compared to other L2s (*d* = 2.16). However, we found that the mean score on the L1s is slightly lower on BibleVocab (80.3 [79.4, 81.2]%) than in WikiVocab (88.3 [87.5, 89.1]%), which is likely caused by the minimal preprocessing, small size, and the use of old and less familiar words in Bible.

Given the minimal curation of Bible texts, one might expect lower discriminability for typologically distant languages, such as Chinese (Sino-Tibetan) and Finnish (Uralic). However, we found no evidence supporting this concern: Chinese participants achieved 73.6 [71.0, 76.2]% on their L1 and 53.2 [51.1, 55.3]% on L2s (*d* = 1.85); Finnish participants scored 80.4 [77.9, 82.9]% on their L1 and 54.5 [51.1, 58.0]% on L2s (*d* = 2.14).

Together, these results suggest that while we have not yet tested all 1,939 languages, both WikiVocab and BibleVocab are likely to generalize well across typologically diverse languages and writing systems.

Meanwhile, differences in vocabulary specificity, level of preprocessing of the source materials and number of tested items may contribute to the observed variation in test–retest reliability between the two instruments. BibleVocab showed lower reliability (*r* = 0.68 [0.63, 0.72]) compared to WikiVocab (*r* = 0.78 [0.75, 0.81]; see Fig. 5*C*). Reduced performance may also stem from misclassified or outdated items—for instance, words like “hash” or “twinkle,” which may appear in modern English but are absent or used differently in biblical texts.

One way to reduce false positives and negatives is to use item selection, namely, by presenting the same test items to a larger pool of participants and selecting those that allow distinguishing between the majority of participants. Item selection can significantly improve the test reliability while also avoiding other issues that our approach does not currently cover. If we want to avoid running an additional experiment, we can also use item repetition instead. Namely, to show the same item twice and measure response consistency. The hypothesis is that nonnative participants are less consistent in their choices because it is difficult to remember their previous response to the same item. Native speakers, in contrast, should be able to consistently determine the lexicality of an item. To test this hypothesis, we relied on the item-repetition design of our experiment, in which each item appeared twice for each participant. An advantage of this approach is that it does not require prior knowledge of whether an item is a real or pseudoword, since scoring is based solely on the consistency of repeated responses. We found that native speakers are significantly more consistent in their lexical decisions than nonnative speakers (native: 92.2 [91.5, 92.8]%, nonnative: 76.2 [75, 77.5]%, *d* = 1.26) and BibleVocab (native: 91.3 [90.6, 92.0]%, nonnative: 74.9 [73.6, 76.3]%, *d* = 1.22) (Fig. 5*D*). This suggests that item repetition can be used as an alternative method to evaluate test reliability, even if some items are mislabeled.

Thus far, we have compared WikiVocab exclusively with other vocabulary tests (e.g., LexTALE and BibleVocab), but not with instruments targeting broader linguistic competencies. To address this, we examined the relationship between WikiVocab and performance in the DIALANG test battery (60), which assesses listening, reading, grammar, vocabulary, and writing skills. Among L2 speakers of Greek (Graeco-Phrygian), Finnish (Finnic), German (Germanic), and Spanish (Italic), WikiVocab scores showed positive correlations with DIALANG performance (*r* = [0.49, 0.64]). These values are comparable to the correlations observed among the individual DIALANG subtests themselves (*r* = [0.64, 0.78]).

These results suggest that vocabulary knowledge, as assessed by WikiVocab, reflects broader linguistic competence. Importantly, this relationship holds across typologically diverse languages, including Greek, Finnish, German, and Spanish, indicating the cross-linguistic robustness of the measure. This pattern of correlations is particularly noteworthy because WikiVocab was not designed to measure these skills directly. Together, these results support the use of WikiVocab as a practical and scalable tool for assessing language proficiency, particularly in contexts where more comprehensive testing is not feasible.

## 2. Discussion

In this study, we introduced a fully automated pipeline to create vocabulary tests for an open-ended number of languages. We constructed two versions of the test: one built from Wikipedia articles in 60 languages and the other based on Bible texts in 1,939 languages. These versions show the range of reliance on online resources. For Wikipedia, we used online dictionaries and a vast amount of text, while for the Bible, we used one single book without extensive online materials. This demonstrates that our method can be applied to any language if there is a sufficiently large collection of texts (for example, the Bible).

With three extensive online experiments, we validated the vocabulary tests in 34 languages. First, we demonstrated that our proposed pipeline can generate vocabulary tests capable of differentiating native speakers from those of closely related languages. Second, we showed that the generated vocabulary tests work for a large number of typologically different languages. Third, we showed that our vocabulary test correlates with a test battery assessing other linguistic competences (e.g., listening and writing). Finally, we demonstrated that the pipeline remains effective even when applied to smaller-sized text corpora and with less preprocessing of the source material (even for character-based and typologically varied languages). Importantly, these results indicate that the general pipeline is robust to known inaccuracies in individual test items, supporting its use as a scalable and reliable proxy for language proficiency.

Importantly, by examining the linguistic background of speakers in their first and second language across 34 languages and countries, we showed that the test score is best predicted by language proficiency self-reports, linguistic classifications, and lexical distance between native and tested languages by using linear mixed-effects models. In general, participants exhibited increased test performances in closely related languages (Fig. 4*B*, e.g., Slovakian–Czech, Russian–Ukrainian), but the test outcome is better predicted using participants’ self-report. This is consistent with the idea that demographic and cultural factors influence the languages people speak [such as their educational system, travel, work, and migration, (61)], and language similarity cannot capture these factors. The results also indicated high reliability in participants’ self-reported language proficiency, although being recruited online.

One might argue that objective testing is unnecessary if individuals can accurately self-report their language proficiency. However, participants in online experiments may be unreliable or may even have incentives to misrepresent their abilities (28, 30). Although the reliability of our test varies across languages, it contributes to the broader set of tools available for assessing language proficiency, particularly in cases where participants have a reason to report inaccurately. Rather than replacing self-report measures, our test is best viewed as a complementary, objective signal that can be deployed at scale. Our test also reveals some variability in performance; however, our results demonstrate that it is effective and reliable in most languages. Moreover, we illustrated how test consistency can be improved through an item-repetition strategy. In the future, performance could be further enhanced using an item-selection approach, where an initial experiment is employed to identify the most reliable items. In any case, we do not imply that our test can be used blindly, careful testing, like we did in this paper, is indeed important, as a variety of factors can affect the test reliability.

While years of formal education are generally strongly correlated with language proficiency self-report (*r* = 0.89 [0.89, 0.90]), it had less predictive power compared to the other three predictors (namely proficiency self-report, linguistic family, and lexical distance). In part, this can be explained by the fact that language skills are also acquired in less formal settings (e.g., in the family, during travel), and not all languages taught in school will lead to a higher language proficiency years after. We also included writing system as a predictor, though it only partially explains the test performance. Writing systems pose an obvious barrier to participants’ performances in a second language. For example, Polish shares some words with Russian, but if a participant cannot read Cyrillic, they should perform at chance level. However, the writing system is generally a rather weak predictor: it fails to predict differences within the same writing system (e.g., a German participant should be better at English than at Finnish), and people can read in multiple writing systems (e.g., many pupils in Poland learn Russian in school and thus can read the Cyrillic alphabet).

By applying our pipeline to the Bible, we showed that our test remains effective when built from much smaller corpora with minimal preprocessing. Despite higher error rates in BibleVocab, we have shown that the test still allows us to distinguish native from nonnative speakers (even in character-based and typologically varied languages, such as Chinese and Finnish). While BibleVocab yields slightly lower native-speaker accuracy than Wikipedia-based tests, this trade-off is highly asymmetrical: A modest reduction in performance enables coverage of nearly two thousand low-resource languages. This substantially expands the ability to screen participants and stratify samples at a global scale, including populations that would otherwise be excluded from language-based research. We therefore aimed to keep the pipeline modular and as language-agnostic as possible.

However, in certain scenarios, e.g., using lab-specific vocabulary tests, the generality of our pipeline comes at a price: It does not take into account every possible orthographic or grammatical rule in all languages. For example, our pipeline produces lowercase nouns in all languages, even though the first letter of German nouns should be capitalized. Another example comes from Hebrew, where we did not remove prefixes or suffixes that are integral to the word (such as the Hebrew letter “Vav” and “Yod”), though a domain expert creating the test would likely exclude them (because they can confuse native speakers in a similar way to typos, which we do eliminate from our test). While experimenters can use our vocabulary tests as a starting point and then apply language-specific rules if needed, we chose not to implement such rules here to preserve maximum cross-language compatibility.

The decision to prioritize either the generality of our pipeline or its sensitivity to language-specific rules depends on how the test is used. We designed our test to distinguish between native speakers of a language and those of closely related languages. In this context, it is acceptable for a subset of the pseudowords not to follow certain rules in a language as these rules are usually not known by less proficient speakers or give no information about the lexicality of test items (e.g., all words are lowercase). However, for lab-based studies, the trade-off might be different. Our approach supports this flexibility by separating automatic item generation from optional, post hoc filtering based on explicit linguistic criteria. This is particularly important for multilingual projects because a) our test exists in a large number of languages and can easily be extended to new languages, b) all items are generated from the same language-agnostic rules, and c) linguistic rules can be specified before filtering which removes the subjectivity inherent to LexTALE and its variants.

Our test targets a specific aspect of linguistic ability—namely, vocabulary knowledge—and does not assess other important dimensions such as grammar, pragmatics, or spoken language skills. Evaluating these additional components would require substantially longer and more complex testing procedures. Our goal, instead, was to develop a psychometrically reliable and time-efficient tool for estimating language proficiency. Despite its narrower scope, we find that performance on our vocabulary test is positively correlated with a comprehensive battery of linguistic assessments—including listening, writing, reading, grammar, and active vocabulary—for four typologically diverse languages: Greek, Finnish, German, and Spanish. These findings suggest that vocabulary knowledge, as measured by WikiVocab, can serve as a meaningful proxy for broader linguistic competence.

Furthermore, the generality of the pipeline makes it applicable beyond the assessment of everyday language proficiency. Because the test items are derived directly from a target corpus, the same infrastructure can be used to construct vocabulary tests for domain-specific or technical jargons, such as medical terminology, legal language, scientific disciplines, or occupational vocabularies. In such cases, real words can be sampled from specialized corpora (e.g., textbooks, manuals, academic articles, or professional guidelines), and pseudowords can be generated using the same language-agnostic principles. This enables the creation of objective tests that distinguish experts from nonexperts, or assess familiarity with specialized domains, without requiring manual curation by domain experts.

Also, the test can provide useful insights in the context of highly multilingual societies, where language proficiency self-report deviates from Euro-American concepts of language proficiency [e.g., a relatively small number of spoken languages per country and high literacy, (62)]. Future research can develop an oral version of the language test, for instance, by utilizing text-to-speech models that are also becoming increasingly available for low-resource languages (63). This would make it possible to extend the pipeline to test participants in less literate societies.

Based on the replication of our findings across Prolific and Cint, we showed that Cint participants tend to be more diverse than those on Prolific. For example, they show less fluency in English than Prolific participants from the same country, indicating that the platform reaches participants from broader social and educational backgrounds. Indeed, we found two demographic differences between both populations: the population on Prolific is relatively young and highly educated, whereas the participants on Cint have a lower overall formal education (Fig. 3*C*, bootstrapped, percentage highest degree is postgrad or higher, Cint: 15.5%, Prolific: 26.2%, *P*< 0.001) and span a larger age range (Fig. 3*D*, bootstrapped, mean age span on Prolific: 41.5 y, Cint: 53.5 y, *P*< 0.001). To reduce the widespread recruiting bias toward participants who are more educated (19, 20), it is thus important to recruit more diverse populations from a wide range of countries from all over the world. Our test enables this research by providing a crucial tool: a validated and scalable language proficiency test across a large number of languages.

Our test can also serve as a tool to monitor data quality. For instance, test–retest reliability can be used as a proxy for the data quality of a participant pool. Our test is also fairly robust against bots. However, Large Language Models and their recent extensions to audio and vision pose a threat to online experiments (64), although recent models frequently misidentify pseudowords as real words (65). We try to address the simple use of language models by introducing the test items as images, though this approach may not deter malicious participants who might utilize multimodal foundational models like GPT-5 in the future (66). Fortunately, there is already substantial knowledge in the literature and industry on distinguishing human participants from bot responses [e.g., Captcha; (67)], so it is necessary to integrate these techniques with our method to ensure that participants do not cheat by employing machine learning bots.

Taken together, we introduced a largely language-agnostic and automated approach to create vocabulary tests using only text. In contrast to existing vocabulary tests, this does not require extensive testing for each language, manual labor by domain experts or the existence of word-frequency databases. This allows us to create a vast number of items of similar quality (see test reliability) that cover the first language of most speakers around the world. Both vocabulary tests can reach a large number of speakers: With WikiVocab, we can reach at least 4.49, and BibleVocab, at least 6.32 billion speakers, which is the far majority of the world population. Our test is available on https://vocabtest.org and is compatible with all modern web browsers and end devices (computers, smartphones, and tablets). To demonstrate the applicability of the tool, we used it to survey language proficiency in 34 languages and across 34 countries. We observed variations in the proficiency of global languages such as English and Spanish, and asymmetries in pairs of languages where L2 speakers are more proficient in one language compared to the other. These variations highlight the impact of complex cultural and demographic factors on language proficiency beyond typography. These findings showcase the usability of our language test both within and outside the field of language studies.

To facilitate broad adoption of the tool, we provide a summary table reporting overall test–retest reliability and mean accuracy in their native and other languages (*SI Appendix*, Table S8). In the experiments reported here, each test block contained 30 items, which offered a practical balance between reliability and administration time. However, this choice is not prescriptive: Researchers may adjust the number of items depending on their goals, bearing in mind the trade-off between finer-grained proficiency estimates and participant time.

In general, researchers may prefer WikiVocab for languages where it is available, as it offers slightly higher accuracy. However, BibleVocab is available in a dramatically larger number of languages, with only a modest reduction in empirical performance. Depending on their goals, experimenters can therefore choose between coverage and accuracy, and can also adapt the method to domain-specific jargon or vocabulary—for instance, by training a corpus of music-theory texts to assess knowledge of musical terminology. We also encountered some language-specific issues in the pipeline. For example, rendering text as images can introduce errors in languages such as Farsi, where context-dependent letter forms may display correctly in the browser but fail when converted to images. These issues were quickly identified and resolved with the help of native speakers, typically by using language-specific text layout libraries. When possible, we recommend consulting native speakers, as this proved highly beneficial during the development and execution of our experiments.

More broadly, our work demonstrates how automatic pipelines can be used to develop research tools for studying humans who speak diverse languages. Several studies have shown that cognitive, psychological, and linguistic research is overly reliant on English and a handful of other majority languages (7, 19, 20). As a result, we may wrongly attribute biological mechanisms to what is specific to some languages, leading to a severely flawed understanding of human behavior. Our work underscores the creation of universally applicable assessment tools, enabling the study of participants beyond just the Western, Educated, Industrialized, Rich, and Democratic (WEIRD) societies (20). Our work demonstrates how a fairly simple, language-agnostic, and extendable pipeline can provide a research tool that can be used to study participants from a large number of linguistic backgrounds.

## 3. Materials and Methods

### 3.1. Participants.

All participants provided informed consent and were recruited through Prolific (52) and Cint Marketplace. The study was approved by the Max Planck Ethics Council (#2021_42). The recruitment and experimental pipelines were automated using PsyNet (68), a framework for automatic experiment design and deployment which builds on the Dallinger (69) platform for recruitment automation. Participants on Prolific received at least 9 GBP. Cint, a recruiting auction that connects researchers with numerous local recruiters worldwide, manages payments that depend on different recruiting companies rather than the researchers directly. However, we endeavored to match participant compensation to the local minimum wage as closely as possible. Overall, we recruited 4,137 participants.

### 3.2. Experiment 1.

Each participant did LexTALE and WikiVocab in their native language and in one of the other seven foreign languages selected at random. The order of the languages is random. For each test and language, there are two blocks of each 30 items. For comparability with the LexTALE tests, we only included the first 60 items in the test, indicating that all people saw all items in the two languages and tests exactly once.

303 participants were recruited through Prolific (*SI Appendix*, Table S2). Our recruitment criteria (based on self-reports) included being born and living in the targeted country, having been raised monolingual, holding nationality from the country, and speaking the target language at home. Since it is not possible to recruit enough participants in Mainland China and there are only a few Finnish participants living in Finland, Chinese, and Finnish participants did not have to reside in their country. Participant numbers varied somewhat due to differences in recruitment difficulty between countries. The goal was to recruit about 40 participants, but we ended up with more or less (*SI Appendix*, Table S2) stopping recruiting after about 24 h. The target number of 40 participants was selected based on preliminary pilot data collected in English so that it matches the typical number of participants in LexTALE validation studies (e.g., ref. 32).

430 participants were recruited from Cint (*SI Appendix*, Table S3). We collected data for the same language-country pairs as on Prolific (e.g., Spanish in Spain and not in Mexico).

### 3.3. Experiment 2 (Language Proficiency Survey).

In total, 2,823 participants from 34 countries participated in the experiments (*SI Appendix*, Table S4). Before the main experiment, participants were asked how well they spoke each of the 34 languages and if they learned it in school. Participants were always tested in their first language and in three randomly chosen languages. If participants indicate they speak other languages beyond their first language, we prioritized them (namely, these languages were also tested if they overlap with our list of 34 languages). This is because if three out of four languages are completely unknown to the participant, performance should be at a chance level, and the task would become random for the participant. For languages that are spoken in multiple countries, we prioritized countries with a larger participant pool. Again, because of the difference in recruiting difficulty, the number of participants somewhat varied from location to location (*SI Appendix*, Table S4), and again we typically recruited participants within about a 24-h window.

We initially included the Urdu test but later excluded it after discovering a display issue affecting the script’s writing order.

For Hebrew, Hindi, and Norwegian, performance among the expected L1 group was lower than that of the other L2 groups.

As a control, we replicated the experiment on Prolific, recruiting native speakers of the target languages (*SI Appendix*, Table S5). Participants were not required to be monolingual (e.g., monolingual Hindi speakers are relatively rare) or to reside in a specific country.

### 3.4. Experiment 3.

We recruited 322 participants for this experiment (*SI Appendix*, Table S6). Each participant does BibleVocab and WikiVocab in their native language and in one of the other seven foreign languages selected at random. The order of the languages is random. For each test and language, there are two blocks of each 20 items. The items are presented at the end of the block again in a randomized order. We used the same demographic filters as in Methods 3.2.

### 3.5. Experiment 4.

We recruited 180 participants for this experiment (*SI Appendix*, Table S7). Each participant does WikiVocab and the full DIALANG test (listening, reading, grammar, vocabulary, writing) (60) in a language they speak, which is not their native language. For each test, there are two blocks.

### 3.6. Statistics.

We use Pearson correlations to measure consistency across tests or blocks of the same test. CI are obtained via bootstrapping (*n* = 1,000).

We used a paired Wilcoxon signed rank test to compare if the difference between the two groups was significant (e.g., WikiVocab score on native language vs. nonnative languages). We report the Cohen’s *d* for each comparison.

## Supplementary Material

Appendix 01 (PDF)

## Data Availability

All code and required data for replication are available on https://github.com/polvanrijn/VocabTest (70). All other data are included in the manuscript and/or *SI Appendix*.

## Footnotes

^*^All code is available on: https://github.com/polvanrijn/VocabTest.

^†^All tests can be administered online: https://vocabtest.org/.

^‡^https://github.com/polvanrijn/VocabTest/tree/main/vocabtest/bible.
