# Supplementary material for: One test, many tongues: Surveying language proficiency across the globe
Source: Proc Natl Acad Sci U S A. 2026 Mar 27;123(13):e2420179123. doi: 10.1073/pnas.2420179123 (PMC13038065; doi:10.1073/pnas.2420179123)
Supplement: Supplementary file 1 — Appendix 01 (PDF) [file pnas.2420179123.sapp.pdf]

Supporting Information for

One Test, Many Tongues: Surveying Language Proficiency Across the Globe

Pol van Rijn, Yue Sun, Harin Lee, Raja Marjieh, Ilia Sucholutsky, Francesca Lanzarini, Elisabeth André, Nori Jacoby

Nori Jacoby.
E-mail: nori.jacoby@ae.mpg.de

This PDF file includes:

Supporting text
Fig. S1
Tables S1 to S8
SI References

Supporting Information Text

A. Difference between Prolific and Cint recruiters. Recruitment data provided by the recruiting services were used to estimate the number of expected participants in the two recruiters. On the 2nd of June 2023, we counted the number of active participants on Cint and on Prolific. For each language, we only include participants living in the country in which the language is spoken. The number of recruitable countries in both recruiters is depicted in the main Figure 3A and the number of languages in main Figure 3B. We also investigated demographic data in the two recruiters. As shown in main Figure 3C–D, participants on Prolific had an overall higher formal education and covered a smaller age span on average than participants on Cint.

B. Selection of Wikipedia articles. We have originally considered various ways of sampling Wikipedia articles. Initially, we considered sampling the articles on the same topic to equate them across the vocabulary tests. However, there is only a limited number of articles on the same topic that exist for all included languages. This constraint would lead to too few articles per language. Another concern is that the length per concept strongly differs across languages (e.g., Finnish Wikipedia might have a long article about Finland, whereas the articles in other languages are shorter). To obtain a large enough vocabulary we ranked all Wikipedia articles in a language by the article length and processed the longest 10,000 articles. For three languages (Gothic, Northern Sami, and Wolof), there were less than 10,000 articles. Since article length differs per language, we include the first 100,000 longest articles if we had less than 5 million valid words to reliably estimate the word frequency of infrequent words occurring down to only once per million.

C. Wikipedia data sources. Wikipedia has been used for various research projects ranging from a database of notable people (1) to studying governance in online communities (2). Due to its size, it is a suitable text source for estimating vocabulary frequency in multiple languages. For each language, we downloaded all Wikipedia articles using 'wiki40b'(3) and 'wikipedia'(4). Both packages implement basic pre-processing, e.g., removing non-content sections. We have originally considered various ways of sampling Wikipedia articles. The selection procedure is described in "Selection of Wikipedia articles" of the SI Appendix.

D. Bible data sources. The Bible is one of the world's most translated texts, making it a good textual source for creating vocabulary tests despite its relatively small size. Bibles were downloaded from https://www.bible.com/. This website includes Bible translations for 2,068 languages. However, some languages only consist of a small portion of the Bible (e.g., only a few chapters) and thus are limited in the number of words. We removed languages that produced a final vocabulary list of less than 30 items, resulting in 1,939 languages.

E. General text-cleaning. The following processing steps are general and are applied both for Wikipedia and the Bible:

General pre-processing. To improve the quality of the generated words, we implemented the following checks: (i) we rejected tokens that are written in exclusively capital letters since they are potential acronyms or anomalies in the text, (ii) we excluded one-letter words in the Latin alphabet as they tend to be used as indexes (e.g., "may n be the number of participants"), and (iii) we removed words containing digits or punctuation. All words are then changed to lowercase.

Remove words with foreign characters To avoid typos, foreign words, or proper nouns, we removed words that contain characters that are not part of the writing system (e.g., a Chinese character in an English word). The removal is done using Unicode, a standardized text encoding to support most of the world's writing systems. Characters in Unicode are organized into blocks. For example, there is a block for Cyrillic, Arabic, or Hebrew characters. We obtained a histogram of Unicode blocks for all characters in each of the accepted tokens. Based on this distribution, we removed words that are not part of the writing system. For WikiVocab, we manually identified the Unicode blocks (based on the expected language). We kept including the largest Unicode blocks for the Bible texts until the cumulative percentage exceeds 50 %.

Detect compound words Compound words are made by combining multiple existing words in a language. Languages differ in their usage of compound words. Certain languages, such as Dutch and German, allow to spontaneously create new words by combining two existing words. This is problematic in the context of the test for two reasons: First, compound words tend to occur much less frequently than each of its components, however, the infrequent compound word is not more difficult than each of the components. This means that compound words are likely to be selected as difficult words, whereas they are not. Second, without removing compound words, the created pseudo-words are more likely to be compound words themselves. Since the text corpus will not contain all possible compound word combinations, the generated word is likely to be marked as a fake word where actually it is an uncommon but real compound word.

Therefore, we trained `charsplit` (5) for each language, which is a model to detect likely word boundaries. The model is trained on all cleaned words. We considered a word to be a compound word if the boundary is likely (> 0, threshold proposed by the author) and if the last segment is a valid word (part of the word list) in that language. We did not use compound word detection for CJK languages (Chinese, Japanese, Korean), as each word is a chain of different characters, and most characters can occur in isolation, so almost all words would be flagged as compound words.

Character to letter conversion For both tests, we converted characters to a letter-like representation from which we can obtain n-grams. In WikiVocab, we used custom packages to convert CJK languages to a letter-like string. For Chinese, we use Pinyin (6), Hiragana for Japanese (7), and Jamo for Korean (8). For the Bible, we checked if the median word length was less than four characters. If this was the case, we assume it is a character-based language, and we converted the characters to Roman

letters using uroman (9). We store the mapping between characters and letters to convert the letters after the sampling back to characters.

F. Wikipedia text-cleaning. The following processing steps were applied only to Wikipedia data. Here, we used tools available for the 60 languages but may not be available for low-resource languages. If the language does not have this resource, this process can be skipped.

General pre-processing In addition to the cleaning procedure described in Methods E, we use the 'isalnum' function in Python to filter alpha-numeric strings (note that this is not supported by all writing systems, e.g., Sanskrit, in only 6 of the 60 languages for which we created the task this function was not available).

Avoid jargon Jargon words such as 'hippocampus' are problematic because they occur infrequently, but they are not known to all native speakers (only known to domain experts). So to avoid marking jargon words as real difficult words, we removed them automatically. To detect possible jargon words, we computed the ratio between the number of occurrences of a word and the number of articles the word occurs in. Jargon words tend to be used frequently in a small number of articles. We, therefore, only kept tokens in the 95 % percentile of the ratio.

Lemmatizer and POS-Tagger Proper nouns are problematic in the context of the vocabulary test since they are names and not words. A POS-tagger labels the Parts Of Speech of all words in the sentence. To make the test more comparable across languages, we only select nouns. We also use a lemmatizer to find the lemma of a given token (e.g., "shoes" → "shoe"). Lemmas are better suited for a vocabulary test than tokens since irregular word forms of tokens can obfuscate the lexical item. Sometimes the lemmatization can lead to a misspelled word form (e.g., "ponies" might be lemmatized as "poni", where "pony" would be the correct lemma). We, therefore, only include lemmas which also exist as tokens. There are various POS-taggers, such as Natural Language Toolkit (10) or 'spacy' (11); however, they mainly support English and a limited set of majority languages. We, therefore, use UDPipe 2.0 (12), which supports more than 60 languages and provides additional meta-information, for example, if the token is a foreign word, an abbreviation, or if it is a typo. We used this tool since it is available in many languages.

Spellchecker The generated pseudo-words should follow the regularities of the language. It is, therefore, key to only include typical (i.e., no foreign words) and correctly spelled words. We use the multilingual language-embedding model `fasttext`, which was trained on 176 languages (13), to predict the language from a given word, which filters out most foreign words. Where available, we use open-source dictionaries from LibreOffice(14) to assess if the word is correctly spelled using the Python packages `guess_language-spirit`(15) and `pyenchant`(16). Certain languages were too close to other languages, leading to a wrong prediction. For example, Western Armenian was flagged as Armenian for 88 % of the tokens. In total, for 13 languages, we could not detect the targeted language in at least 35 % of the tokens. In these cases, we did not exclude words based on the spellchecker. The 13 languages are Faroese, Irish, Scottish Gaelic, Galician, Gothic, Western Armenian, Latin, Maltese, Norwegian Nynorsk, Sanskrit, Northern Sami, Uyghur, and Wolof.

G. Bible text-cleaning. The following processing steps were only applied to the Bible data. We explicitly did not rely on language-specific resources like dictionaries that are not available in all Bible languages. We first align all verses overlapping with an English reference Bible ("New Living Translation") using a universal text alignment tool `fast-align` (17). We used the Spacy POS tagger to mark proper nouns (11) and searched for proper nouns in the English translation from a curated list(18). We then aggregated over all occurrences of a stopword (e.g., "Israel"). For each reference stopword, we retrieved all aligned target words. Since the alignment is not perfect, the same stopword is not always aligned to the same target word. We only included the most common target word if it's used in more than 20 % of the alignments. Since the word might be spelled slightly differently due to inflection, we included words in the stop list that are similar to the top match (> 80 % fuzzy match). We excluded those stopwords obtained from the previous procedure to reduce proper nouns in the translations. For all languages without character conversion (Method E), we used compound word detection (Methods E) and removed words of an untypical length.

H. Pseudo-words generation. Both WikiVocab and BibleVocab used the following steps.

Compute n-grams Existing linguistic work on spoken lexicons of multiple languages has shown that pseudo-words generated from a 5-phone model capture most phonotactic regularities across the real words of most languages (19, 20). We, therefore, used 5-gram transitional probabilities to create pseudo-words since they are the closest equivalents to 5-phone transitional probabilities for the written language. To track different transition probabilities at the beginning and end of each word, we padded the beginning and end of each word with asterisks (our symbol for word termination).

Sample from n-grams We begin by choosing a sequence of five characters, starting with four asterisks ('****') to signify the word's beginning. For each subsequent sequence, the initial four characters match the final four of the preceding one. This process continues until we select a sequence ending in an asterisk, signaling the word's end. After removing the asterisks, we checked the resulting letter string. Using this padding method, words with fewer than five letters can be created if the termination symbol occurs earlier. We continued this process until we have generated 1,000 unique pseudo-words.

Validate pseudo-words We rejected generated pseudo-words that correspond to real words in the language (tested using our corpus). We also rejected pseudo-words that contain too few or too many letters based on the range of word length of the real words in our list for each language (± 2 SD from the median word length). For character-based languages like Chinese, we convert each pseudo-word from the letter-based representation back to the character representation. We do this by replacing all characters. To replace the longest letter sequences first, we sort the letter-character mapping by the length of the letter string. If not all letters in the pseudo-word can be replaced by characters, the word is rejected. For all other languages, we checked if the created pseudo-words are likely to be compound words (as explained in section E). We rejected the word if this is the case. To avoid the creation of pseudo-words that looked similar to existing words and are potential typos, we compute a fuzzy search using `thefuzz`(21). Since the total number of words is extremely large, we limited the search to words that start with the first and last three letters and are of a similar length (10 % difference in length allowed). We stored the maximum match between the pseudo-words and any word in that language.

I. Select real words. To match the task difficulty of our task to LexTALE, thus making them comparable for experiments, we first identified the real LexTALE items in our word frequency distribution (log10-scale). We then compute the mean and standard deviation of the LexTALE items per language. We select real words by finding words with frequencies that are randomized from a normal distribution centered at the average LexTALE word frequency, with the standard deviation being computed over all languages with LexTALE. The same mean and standard deviation were used for all languages, as well as for languages without LexTALE.

J. Creating pairs or words and pseudo-words with matched difficulty. In the actual tests, it was important to balance the difficulty of real and pseudowords. We, therefore, created pairs of tests with similar expected difficulty and structure. Of course, in the actual experiment, the words were presented in random order, so participants could not take advantage of this pairing. Out of the 1,000 created pseudo-words, we selected 500 that best matched the words in that language. To do so, we obtained the logarithm of the transitional probabilities of the letters' 5-grams for both the words and the pseudo-words. We then computed the average absolute difference between the words and pseudo-words that have the same number of 5-grams. On the resulting distances, we performed greedy matching, where we kept matching the word and pseudo-words with the smallest distance. We then matched words and pseudo-words with a similar 'rarity' at the same position. We repeat this procedure until we match all pseudo-words. From the matched list, we only included 500 matched pseudo-words that have the smallest fuzzy match ratio to any of the words in that language. By doing so, we selected the 500 pseudo-words that are least likely to be typos (as typos are hard to detect even for native speakers, especially when words are presented quickly). This procedure tries to ensure that the low-level statistics of characters of words and pseudo-words are similar, thus preventing participants from using this knowledge to resolve the task without real lexical knowledge.

K. Interface. Words and pseudo-words were presented in random order. To reduce the chance that a participant will search for the word on the internet, we displayed the word as an image (thus, the participant could not copy it as text) and limited the display time to two seconds. Participants were asked to respond as fast as possible by pressing two dedicated keys on their keyboard. To estimate the reliability of the test, participants did two batches of trials per language and test. Each batch contained 30 trials except for the last experiment, where it's 20 trials and 20 repetitions of the same trials. All texts in the interface of the experiment (e.g., buttons, instructions, etc.) were presented in the participant's native language. Non-English texts were automatically translated using DeepL or, if the language was not supported, then by Google Translate.

L. Global coverage of tests. To estimate the number of speakers of a language, we retrieved the number of active speakers from WikiData by searching by a global identifier for families, languages, and dialects (Glottolog, 22). We only include Glottologs with the status of a 'language' and get the total number of speakers for them. We retrieve the countries where the language is spoken from the Glottolog database (23). However, this is a rough estimate since it does not consider multilingual (i.e., double counts), and the speaker estimates are not always up to date.

Supplementary Figures

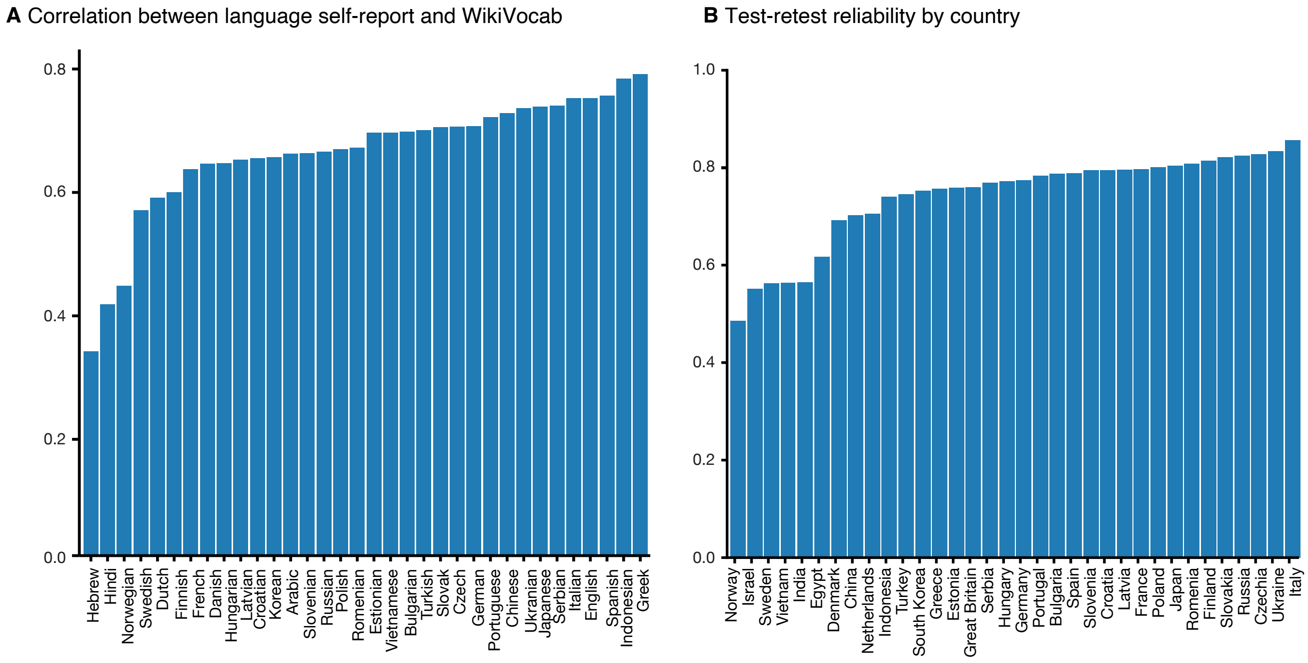

Fig. S1. **A** Correlation between language self-report and WikiVocab. For each country, we compute the correlation between the language self-report and the test performance. We found that for the countries with a weak diagonal, the correlation is the lowest. **B** WikiVocab reliability per country on Cint. We measured the reliability by correlating the test scores of two blocks. Each bar in represents the correlation between the first and second tests in the same language. The reliability was measured on the performance of all languages in a given country and not only on the native language. Again, the countries with the weak diagonal have the lowest test-retest reliability.

Supplementary Tables

Language	Correct	Incorrect
Arabic	متوازي	مثاقين
Chinese	追思	柿且
Dutch	rekruut	kalling
English	collier	versary
French	ossature	pistence
German	zündung	söhnung
Hebrew	מקפצה	נימום
Italian	miriade	intenza
Korean	분말	만중
Russian	лепнина	невозка
Spanish	escorzo	septil
Turkish	zafiyet	balyon

Table S1. Example items for 12 languages.

Language	Country	Count
Dutch	the Netherlands	38
English	Great Britain	39
Finnish	*	35
French	France	39
German	Germany	39
Italian	Italy	38
Mandarin Chinese	*	35
Spanish	Spain	40
		**303**

Table S2. Participants per country on Prolific in experiment 1. * indicates that participants did not have to reside in the country.

References

1. M Laouenan, et al., A cross-verified database of notable people, 3500bc-2018ad. *Sci. Data* **9** (2022).
2. N Schneider, PD Filippi, S Frey, JZ Tan, AX Zhang, Modular politics. *Proc. ACM on Human-Computer Interact.* **5**, 1–26 (2021).
3. Google, Wiki40b (2023).
4. Wikimedia, Wikipedia (2025).
5. D Tuggener, Ph.D. thesis (University of Zurich) (2016).
6. H Huang, Python-pinyin (2025).
7. H Miura, Pykakasi (2025).
8. H Shin, Jamotools (2025).
9. Hermjakob, Ulf, Uroman (2025).
10. E Loper, S Bird, Nltk: The natural language toolkit (2002).
11. I Montani, et al., explosion/spacy: v3.7.2: Fixes for apis and requirements (2023).
12. M Straka, UDPipe 2.0 prototype at CoNLL 2018 UD shared task in *Proceedings of the CoNLL 2018 Shared Task: Multilingual Parsing from Raw Text to Universal Dependencies.* (Association for Computational Linguistics, Brussels, Belgium), pp. 197–207 (2018).
13. P Bojanowski, E Grave, A Joulin, T Mikolov, Enriching word vectors with subword information. *arXiv preprint arXiv:1607.04606* (2016).
14. LibreOffice dictionaries (2025).
15. Guess_language-spirit (2025).
16. Pyenchant (2025).
17. C Dyer, V Chahuneau, NA Smith, A simple, fast, and effective reparameterization of ibm model 2 in *North American Chapter of the Association for Computational Linguistics.* (2013).
18. R Rouse, Theographic Bible metadata (2025).

Language	Country	Count
Dutch	the Netherlands	42
English	Great Britain	80
Finnish	Finland	29
French	France	46
German	Germany	47
Italian	Italy	84
Mandarin Chinese	China	17
Spanish	Spain	85
		**430**

Table S3. Participants per country on Cint in experiment 1.

Language	Country	Count
Arabic	Egypt	146
Bulgarian	Bulgaria	90
Croatian	Croatia	31
Czech	Czechia	80
Danish	Denmark	25
Dutch	the Netherlands	93
English	Great Britain	152
Estonian	Estonia	54
Finnish	Finland	68
French	France	90
German	Germany	111
Greek	Greece	261
Hebrew	Israel	42
Hindi	India	108
Hungarian	Hungary	48
Indonesian	Indonesia	87
Italian	Italy	128
Japanese	Japan	108
Korean	South Korea	34
Latvian	Latvia	74
Mandarin Chinese	China	16
Norwegian	Norway	91
Norwegian*	Norway*	47
Polish	Poland	108
Portuguese	Portugal	112
Romanian	Romania	79
Russian	Russia	43
Serbian	Serbia	53
Slovakian	Slovakia	43
Slovenian	Slovenia	62
Spanish	Spain	99
Swedish	Sweden	30
Turkish	Turkey	118
Ukrainian	Ukraine	40
Vietnamese	Vietnam	52
		**2,823**

Table S4. Participants per country on Cint in experiment 2.

Language	Count
Hebrew	41
Hindi	29
Norwegian	11
	**81**

Table S5. Participants for control experiments on Prolific in experiment 2.

Language	Country	Count
Dutch	the Netherlands	40
English	Great Britain	41
Finnish	*	38
French	France	40
German	Germany	39
Italian	Italy	40
Mandarin Chinese	*	44
Spanish	Spain	40
		**322**

Table S6. Participants per country on Prolific in experiment 3.

Language	Count
Finnish	30
Greek	48
Spanish	43
German	59
	**180**

Table S7. Participants per language on Prolific in experiment 4.

Language	Test-retest reliability	L1 accuracy	Other accuracy
**Arabic**	0.62	69.3 [68.1, 70.6] %	50.0 [49.5, 50.6] %
**Bulgarian**	0.79	86.3 [84.3, 88.3] %	58.1 [56.9, 59.3] %
**Croatian**	0.80	81.5 [77.9, 85.1] %	57.5 [55.6, 59.4] %
**Czech**	0.83	85.3 [83.5, 87.2] %	56.1 [55.0, 57.3] %
**Danish**	0.68	74.9 [70.9, 78.8] %	56.5 [54.4, 58.6] %
**Dutch**	0.71	72.4 [69.9, 74.9] %	58.1 [57.0, 59.2] %
**English**	0.78	83.2 [81.9, 84.6] %	52.4 [51.7, 53.1] %
**Estonian**	0.76	73.6 [70.6, 76.6] %	57.0 [55.5, 58.5] %
**Finnish**	0.81	85.9 [83.7, 88.0] %	55.1 [54.0, 56.2] %
**French**	0.79	80.7 [77.9, 83.5] %	54.3 [53.3, 55.3] %
**German**	0.78	79.9 [77.9, 81.9] %	54.5 [53.5, 55.4] %
**Greek**	0.76	83.6 [82.6, 84.6] %	55.9 [55.3, 56.6] %
**Hebrew**	0.57	57.4 [54.3, 60.5] %	53.9 [52.4, 55.5] %
**Hindi**	0.57	57.2 [55.9, 58.6] %	53.9 [53.0, 54.8] %
**Hungarian**	0.78	84.7 [82.6, 86.8] %	54.4 [53.0, 55.8] %
**Indonesian**	0.74	79.2 [77.5, 81.0] %	52.9 [51.9, 53.9] %
**Italian**	0.86	89.2 [87.6, 90.7] %	57.9 [56.9, 59.0] %
**Japanese**	0.80	84.4 [83.1, 85.7] %	50.7 [50.0, 51.5] %
**Korean**	0.74	75.3 [70.0, 80.6] %	50.4 [49.3, 51.6] %
**Lithuanian**	0.80	80.4 [78.1, 82.7] %	57.6 [56.2, 58.9] %
**Mandarin Chinese**	0.70	74.9 [70.2, 79.5] %	47.9 [45.9, 49.9] %
**Norwegian Bokmål**	0.50	55.5 [53.4, 57.6] %	51.2 [50.3, 52.1] %
**Polish**	0.80	82.7 [81.2, 84.3] %	53.0 [52.2, 53.8] %
**Portuguese**	0.78	83.3 [81.7, 84.9] %	58.7 [57.7, 59.8] %
**Romanian**	0.81	85.1 [83.2, 86.9] %	58.6 [57.2, 60.0] %
**Russian**	0.83	88.2 [86.0, 90.3] %	54.0 [52.7, 55.2] %
**Serbian**	0.77	84.8 [82.8, 86.7] %	57.5 [56.0, 58.9] %
**Slovak**	0.82	83.7 [80.9, 86.6] %	59.1 [57.3, 60.9] %
**Slovenian**	0.79	79.3 [76.8, 81.8] %	58.0 [56.5, 59.4] %
**Spanish**	0.79	86.7 [85.1, 88.2] %	56.0 [54.9, 57.1] %
**Swedish**	0.58	70.7 [67.1, 74.4] %	57.1 [55.2, 59.0] %
**Turkish**	0.75	82.3 [81.0, 83.7] %	52.6 [51.9, 53.4] %
**Ukrainian**	0.83	82.2 [79.0, 85.5] %	61.9 [59.4, 64.3] %
**Vietnamese**	0.57	71.9 [69.5, 74.4] %	52.3 [51.3, 53.3] %

Table S8. Test–retest reliability (Pearson correlation) between two WikiVocab test blocks, and mean test accuracy for native (L1) versus other languages. Values in square brackets indicate 95% confidence intervals.

19. I Dautriche, K Mahowald, E Gibson, A Christophe, ST Piantadosi, Words cluster phonetically beyond phonotactic regularities. *Cognition* **163**, 128–145 (2017).
20. S Trott, B Bergen, Why do human languages have homophones? *Cognition* **205**, 104449 (2020).
21. Thefuzz (2025).
22. H Hammarström, R Forkel, M Haspelmath, S Bank, glottolog/glottolog-cldf: Glottolog database 4.8 as CLDF (2023).
23. H Hammarström, R Forkel, Glottocodes: Identifiers linking families, languages and dialects to comprehensive reference information. *Semantic Web J.* **13**, 917–924 (2022).
